# Glue versus absorbable tacks for mesh fixation in laparoscopic inguinal hernia repair: a systematic review and meta-analysis of postoperative pain and complications

**DOI:** 10.1007/s10029-026-03757-w

**Published:** 2026-07-03

**Authors:** Caio Pluvier Duarte Costa, Ana Paula Valerio-Alves, Rachel Gomes Boechat de Oliveira, Thablu Matheus Alves Gonzaga, Ana Beatriz de Almeida Porto Maia, Zuila Rafaella Cavalcante de Oliveira, Rafael Morriello, Marcelo Henrique Ferreira Fernandes

**Affiliations:** 1 Department of General Surgery, Federal Servants Hospital of Rio de Janeiro, Sacadura Cabral Street, Rio de Janeiro, RJ 17820221-161 Brazil; 2 Department of Medicine, Barao de Maua University Center, Ribeirao Preto, Sao Paulo, Brazil; 3 Department of General and Gastrointestinal Surgery, Instituto de Medicina Integral Professor Fernando Figueira (IMIP), Av. Governador Agamenon Magalhaes, Edifício Joao de Deus - 1º Andar - Paissandu, Recife, PE 4760 Brazil; 4 Department of Oncological Surgery, Brazilian National Cancer Institute, 20231-130, Rio de Janeiro, Brazil

**Keywords:** Inguinal hernia, Laparoscopic repair, Mesh fixation, Surgical glue, Absorbable tacks, Postoperative pain

## Abstract

**Purpose:**

Mesh fixation is not routinely required in most laparoscopic inguinal hernia repairs (LIHR); however, when indicated, the optimal fixation method remains uncertain. This study aimed to compare surgical glue versus absorbable tacks (AT) for mesh fixation in LIHR, focusing on postoperative pain and early complications.

**Methods:**

We searched PubMed, Embase, and Cochrane for randomized controlled trials (RCTs) comparing glue versus AT in elective LIHR. Effect estimates were pooled using random-effects models and expressed as mean differences (MDs) or risk ratios (RRs) with 95% confidence intervals (CIs). The primary outcome was postoperative pain assessed by the visual analogue scale (VAS). Secondary outcomes included complications and operative time. Risk of bias was assessed using the Cochrane RoB 2 tool. Trial sequential analysis (TSA) and prediction intervals (PI) were used to assess robustness.

**Results:**

Nine RCTs comprising 1,289 patients were included. Glue fixation was associated with lower early postoperative pain at 7–14 days (MD − 0.77; 95% CI − 1.22 to − 0.33; *p* < 0.001; I^2^ = 83%). However, substantial heterogeneity and wide prediction intervals (− 2.33 to 0.79) indicated variability across settings. TSA suggested evidence of potential effect; however, this finding should be interpreted in the context of substantial heterogeneity. No significant difference was observed beyond 4 weeks, with insufficient cumulative evidence to draw firm conclusions. Glue fixation was associated with lower rates of hematoma and seroma, although the findings were uncertain given the prediction intervals and TSA. Other outcomes were underpowered or inconclusive.

**Conclusion:**

Glue fixation may be associated with lower early postoperative pain than absorbable tacks after LIHR; however, the available evidence remains heterogeneous and inconclusive for other postoperative outcomes. Further adequately powered randomized trials are required before definitive clinical recommendations can be made.

**Supplementary Information:**

The online version contains supplementary material available at 10.1007/s10029-026-03757-w.

## Introduction

Despite the considerable success of tension-free mesh repair in diminishing inguinal hernia recurrence rates, from approximately 60% associated with traditional tissue approximation to as low as 1% [1], postoperative pain continues to be a significant concern affecting 10–30% of patients in the early postoperative period, whereas chronic postherniorrhaphy neuralgia persists in 0.5–16% of cases [2, 3]. Laparoscopic approaches, including transabdominal preperitoneal (TAPP) and totally extraperitoneal (TEP) repair, have demonstrated comparable outcomes in terms of recurrence and pain [4], suggesting that multiple factors, including patient characteristics and technical aspects of the procedure, may influence postoperative recovery [5]. Among these factors, mesh fixation has historically been considered a relevant technical component.

Although mesh fixation has traditionally been considered an important step in laparoscopic inguinal hernia repair, current guidelines indicate that fixation is not routinely required in most cases, particularly in small or indirect hernias [5]. Instead, fixation is generally reserved for selected situations, such as large direct defects or increased risk of mesh displacement. When fixation is indicated, different strategies are available, broadly classified as penetrating methods (e.g., tacks and sutures) and non-penetrating methods (e.g., adhesives) [6]. While penetrating fixation has been associated with complications such as neuralgia [3] and bleeding or hematoma formation [9], atraumatic fixation techniques, including surgical adhesives, have emerged as potential alternatives to reduce postoperative morbidity [8]. These adhesives degrade over time, allowing progressive tissue integration and reducing reliance on mechanical fixation [10]. However, the comparative effectiveness of these fixation methods remains uncertain, particularly regarding postoperative pain and early complications. Previous studies have reported heterogeneous findings, often influenced by variations in surgical techniques and outcome assessment [6]. In addition, the inclusion of different fixation devices and the lack of high-certainty evidence further limit the interpretation of results [3, 7]. In this context, the most relevant clinical question is not which fixation method should be routinely preferred, but rather which approach may be more appropriate when fixation is deemed necessary. Therefore, this systematic review and meta-analysis of randomized controlled trials aimed to compare surgical glue versus absorbable tacks for mesh fixation in laparoscopic inguinal hernia repair, focusing on postoperative pain and early complications.

## Methods

We performed a systematic review and meta-analysis following the Cochrane Collaboration guidelines and the Preferred Reporting Items for Systematic Reviews and Meta-Analyses (PRISMA) statement. This study was prospectively registered on the international database of systematic review protocols (PROSPERO) under protocol CRD420251017746 *version 2.0*.

### Eligibility criteria

The studies included in this meta-analysis were selected based on the following eligibility criteria: (1) RCTs; (2) LIHR procedures, specifically TAPP or TEP approaches; (3) participants aged 18 years or older; and (4) comparative analysis between AT and surgical adhesives (fibrin or cyanoacrylate-based glues). Studies were excluded if they met any of the following conditions: (1) using non-AT or self-gripping meshes; (2) non-randomized study designs; (3) absence of a control group; or (4) overlapping patient populations across multiple studies. These criteria were established to ensure methodological rigour and minimize bias in the analysis.

### Search strategy and data extraction

We conducted a systematic review in February 2025, using the databases of PubMed, EMBASE, and Cochrane Central Register of Controlled Trials. The search strategy employed the following Medical Subject Headings (MeSH) and keywords: “Hernia repair”, “Glue”, and “Tacks”. The full electronic search strategy, including Boolean operators and database-specific syntax, is provided in Supplementary Table 1. Additionally, the reference lists of all included studies and relevant prior reviews were manually screened to identify potentially eligible articles. Two independent reviewers (AP and RG) performed the data extraction and quality assessment under predefined search criteria. In instances of disagreement, a third reviewer (CP) was consulted to reach a consensus on study inclusion, under predefined eligibility and extraction criteria. When reported, clinical variables such as hernia type, defect size, and indication for fixation were extracted to improve clinical interpretation. This approach ensured a comprehensive and unbiased selection of studies for analysis.

### Endpoints analyzed

The primary endpoint evaluated in this study was postoperative pain, assessed using the Visual Analog Scale (VAS), measured between postoperative days 7 and 14, and at a follow-up beyond four weeks. Secondary outcomes of interest included wound infection, operative time (measured in minutes), hospital length of stay (LOS), hernia recurrence, hematoma formation, and urinary retention. Secondary outcomes were considered exploratory, given the limited number of studies and expected variability in reporting.

### Statistical and sensitivity analysis

Statistical analyses were performed using Review Manager (RevMan) version 5.1 (Nordic Cochrane Centre, The Cochrane Collaboration, Copenhagen, Denmark). For dichotomous outcomes, pooled effects were expressed as risk ratios (RRs), and for continuous outcomes as mean differences (MDs), both with 95% confidence intervals (CIs). Between-study heterogeneity was assessed using the Cochran Q test and quantified with the I^2^ statistic. Pooled estimates were calculated using a random-effects model to account for variability across studies. When continuous data were reported as medians with ranges or interquartile ranges, these values were converted to means and standard deviations using established methods [11, 12]. In addition to 95% confidence intervals, 95% prediction intervals were calculated for key outcomes within RevMan to reflect the expected range of effects in future studies under a random-effects framework.

Additionally, to evaluate the robustness of cumulative evidence and the risk of random errors, a trial sequential analysis (TSA) was performed using Trial Sequential Analysis software (version 0.9.5.10 Beta; Copenhagen Trial Unit, Centre for Clinical Intervention Research, Rigshospitalet, Copenhagen, Denmark), assuming a two-sided α of 0.05 and 80% power (β = 0.20) based on the based on the effect size estimated from the pooled random-effects model. Cumulative Z-curves were assessed against conventional significance boundaries, trial sequential monitoring boundaries for benefit and futility, and the required information size. Sensitivity analyses were conducted using a leave-one-out approach, to explore the robustness of pooled estimates and identify potential sources of heterogeneity.

### Quality assessment

Studies were evaluated using Cochrane’s Risk of Bias 2 (RoB2) tool for RCTs, which assesses five domains: (1) bias arising from the randomization process, (2) deviations from intended interventions, (3) missing outcome data, (4) measurement of the outcome, and (5) selection of the reported result. To assess potential publication bias, given the small number of studies, funnel plot interpretation was considered exploratory. Certainty of evidence was assessed using the GRADE framework.

## Results

### Study selection and characteristics

**Fig. 1:**
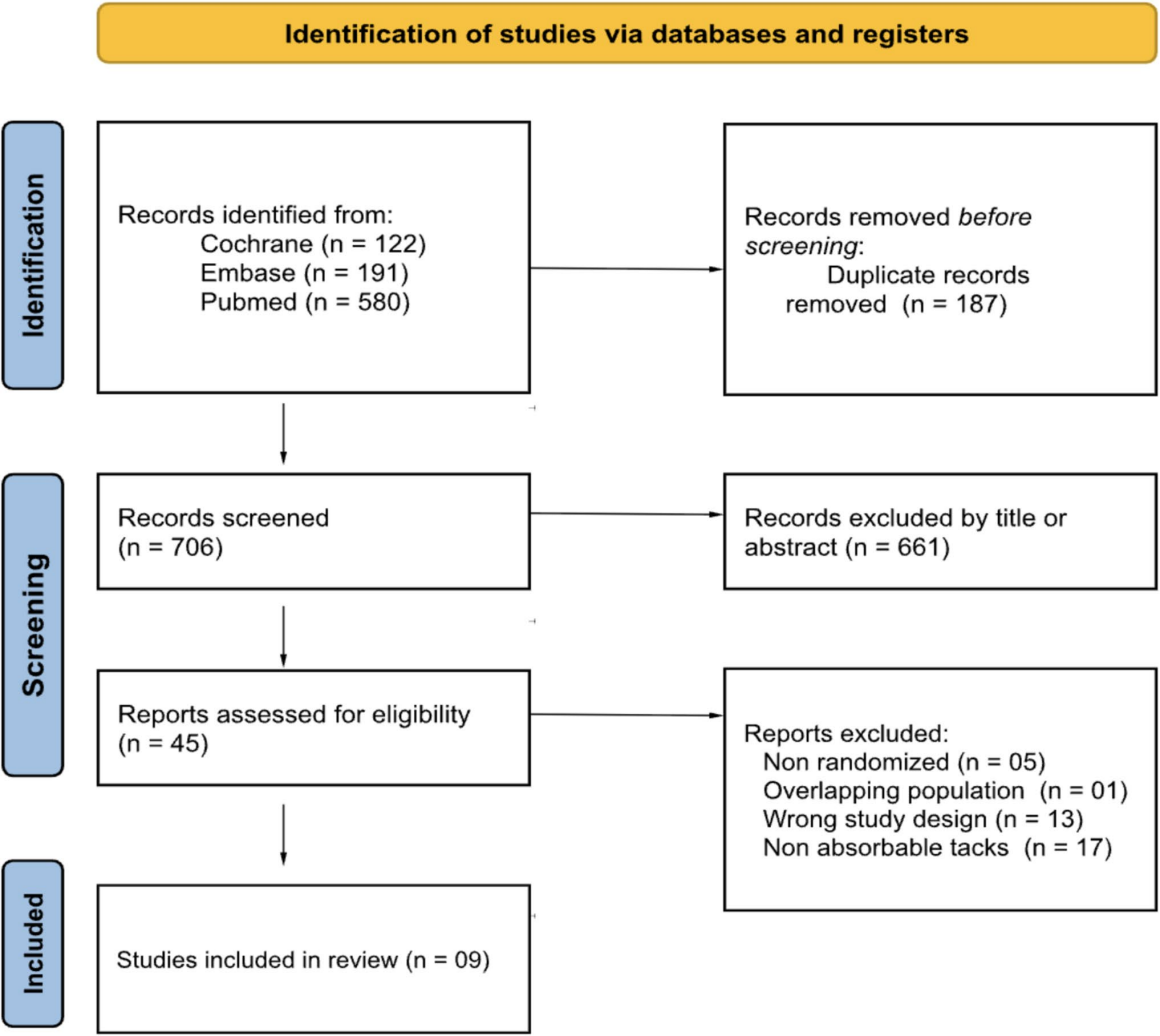
PRISMA flow diagram of study screening and selection

The initial search yielded 893 records. After removing duplicate records and unrelated studies, 39 remained and were fully reviewed for eligibility against the inclusion criteria (Fig. 1). Following the exclusion of studies, 9 RCTs were included with a total of 1289 patients incorporated into the review (Table 1). Glue was used in 651 (50.50%), while AT was used in 638 (49.50%) patients for mesh fixation. The male sex accounted for 988 (89.81%), with an average age of 60.0 ± 9.0 years, and 1084 (94.26%) patients underwent primary hernioplasty. Relevant clinical variables, including defect size and indications for mesh fixation, were inconsistently reported across studies, limiting further evaluation of potential clinical heterogeneity.

**Table 1:** Baseline characteristics of included studies

Study	Year	No of patients	I/C*	No of males (%)	Age I/C* (mean)	Follow-up (months)	Primary hernia (%)	Direct hernia (%)	Indirect hernia (%)	Bilateral hernia (%)	TAPP/TEP approach (%)
Olmi	2007	300	150/150	97.7	44/42	1	22.2	-	-	33.8	100/0
Moreno-Egea	2014	106	52/54	70.7	55.8/54.9	24	9.8	14.1	85.8	100	0/100
Cristaudo	2015	139	81/58	-	-	2	-	-	-	58.9	0/100
Issa	2021	106	51/55	96.2	48.5/57.9	6	9.8	43.3	38.1	-	0/100
Azevedo	2022	42	21/21	100	-	24	3.9	19.0	80.9	0	100/0
Jeroukhimov	2023	208	102/106	93.7	54.4/54.5	12	31.9	26.5	67.3	66.3	0/100
Manish	2023	54	27/27	20.4	-	3	5.0	-	-	-	100/0
Emad	2024	50	25/25	-	38.0	6	4.6	-	-	0	100/0
Petro	2024	284	142/142	95.1	61/59	12	12.8	40.2	59.7	40.4	65.5/34.5

*I/C, intervention/control

### Pooled analysis of all studies

#### Early postoperative pain (7–14 days)

**Fig. 2:**
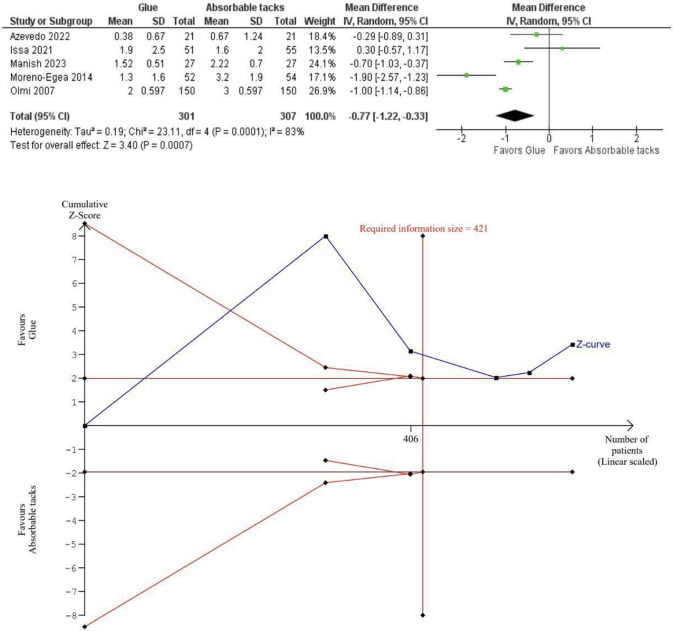
Forest plot and trial sequential analysis of early postoperative pain (VAS, postoperative days 7–14) comparing glue fixation with absorbable tacks in laparoscopic inguinal hernia repair

Five randomized controlled trials (608 patients) assessed postoperative pain between days 7 and 14 (Fig. 2). Random-effects meta-analysis was associated with lower pain scores with glue fixation compared with absorbable tacks (mean difference − 0.77; 95% CI − 1.22 to − 0.33; *p* < 0.001), with substantial heterogeneity (τ^2^ = 0.19; I^2^ = 83%). A leave-one-out sensitivity analysis was performed; however, no single study accounted for the observed heterogeneity, suggesting underlying clinical variability. The 95% prediction interval (− 2.33 to 0.79) indicated variability in effect magnitude across settings. TSA suggested evidence of a potential effect; however, this finding should be interpreted with caution, given the substantial heterogeneity and wide prediction intervals. The clinical relevance of this effect size remains uncertain, given the substantial between-study heterogeneity.

#### Late postoperative pain (≥ 4 weeks)

**Fig. 3:**
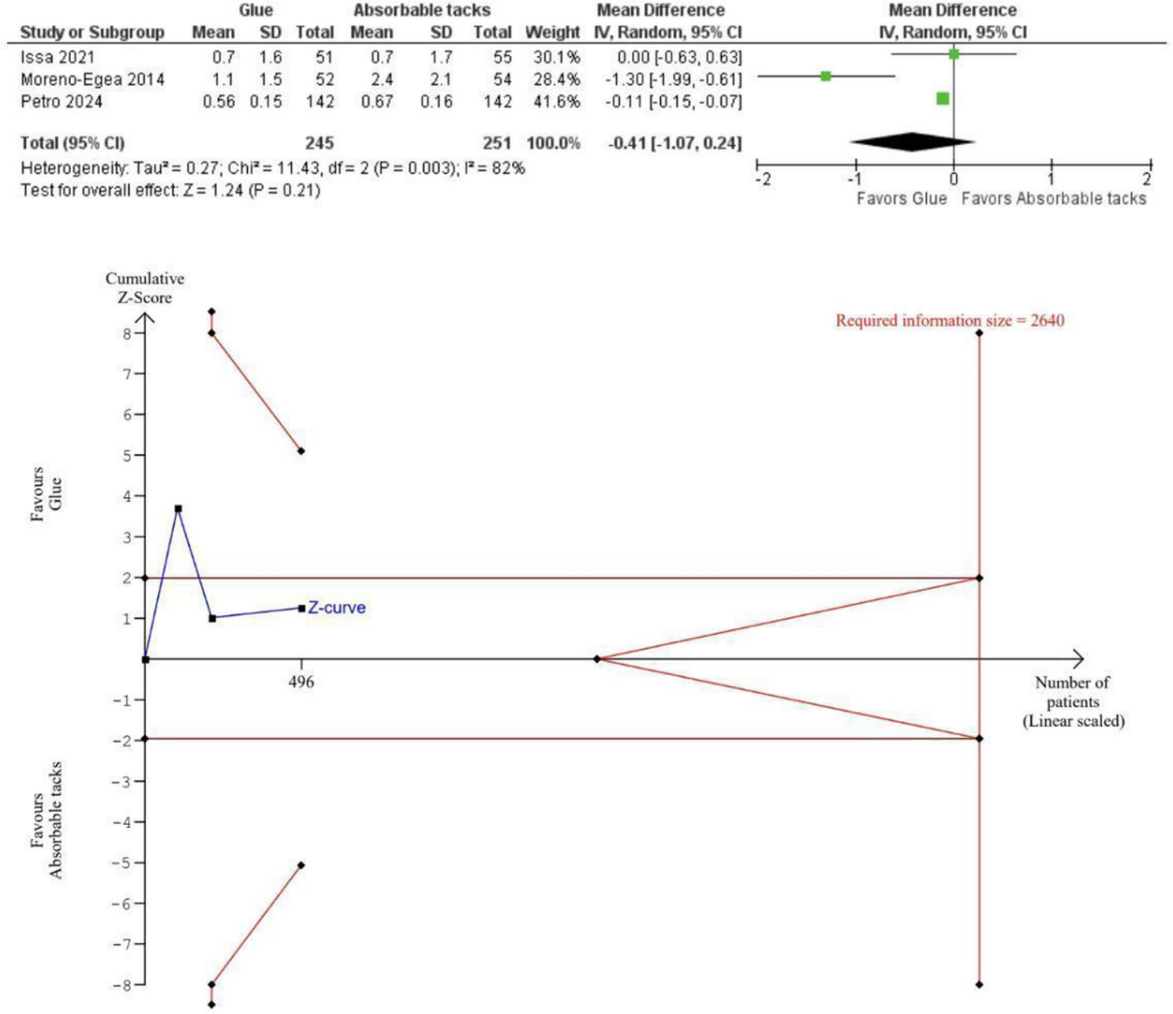
Forest plot of postoperative pain assessed by the visual analogue scale (VAS) at ≥ 4 weeks comparing glue fixation with absorbable tacks in laparoscopic inguinal hernia repair and Trial sequential analysis

Six trials evaluated pain at ≥ 4 weeks; three (496 patients) were pooled because the remaining studies reported VAS scores of zero (Fig. 3). No significant difference was observed (mean difference − 0.41; 95% CI − 1.07 to 0.24; *p* = 0.21), with substantial heterogeneity (τ^2^ = 0.27; I^2^ = 82%). A leave-one-out sensitivity analysis suggested that Moreno-Egea et al. may have contributed to the observed heterogeneity, potentially related to differences in assessment timing (4 vs 12 weeks). After exclusion of this study, the pooled estimate was − 0.11 (95% CI − 0.15 to − 0.07; *p* < 0.05), with no residual heterogeneity (τ^2^ = 0; I^2^ = 0%). However, this finding should be interpreted cautiously, as it results from a post hoc sensitivity analysis and remains exploratory, not reflecting the primary pooled estimate. TSA did not cross significance or futility boundaries and did not achieve the required information size (2,640 patients), indicating insufficient evidence to draw firm conclusions.

#### Secondary outcomes

**Fig. 4:**
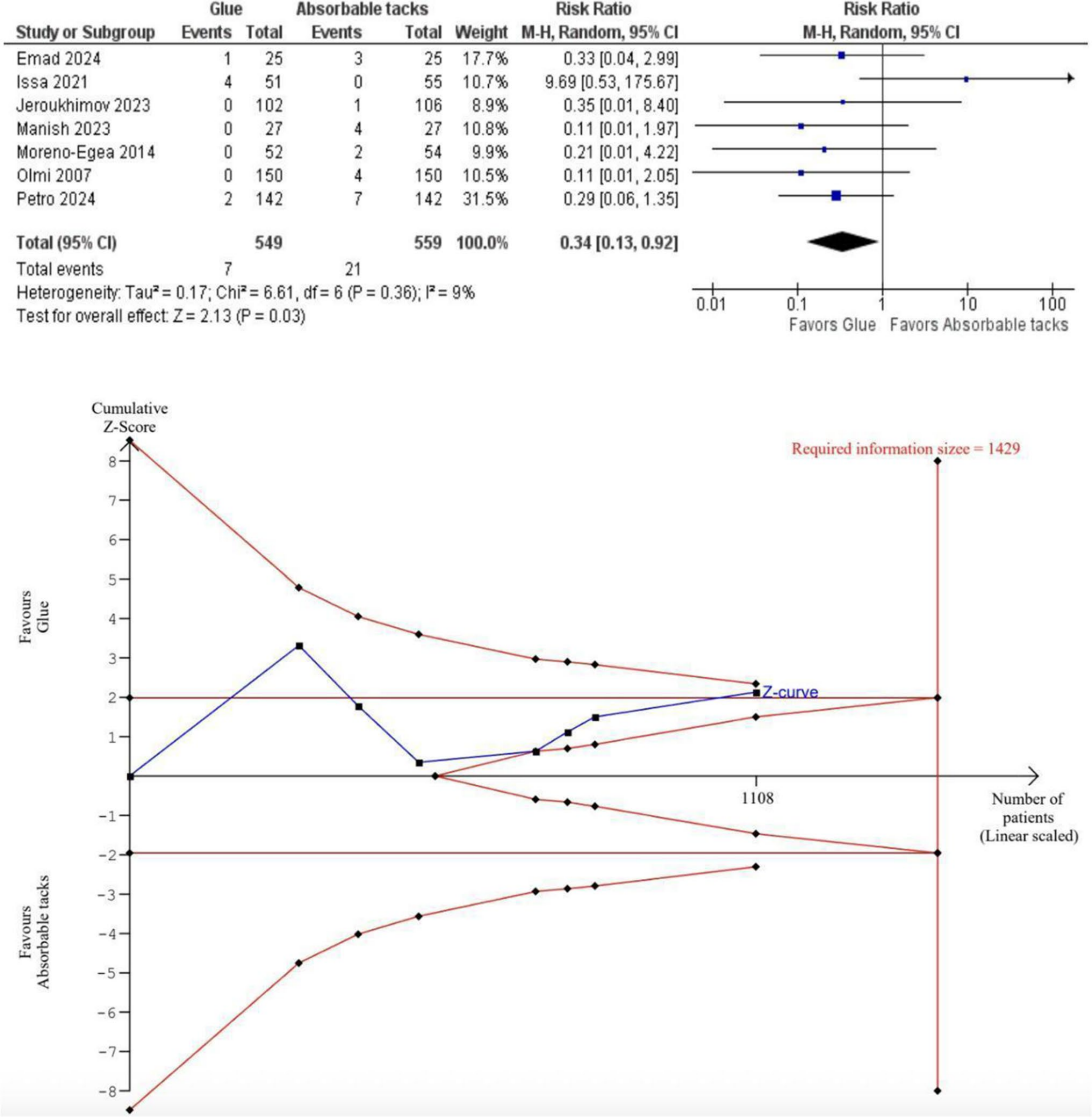
Forest plot and trial sequential analysis of postoperative hematoma comparing glue fixation with absorbable tacks in laparoscopic inguinal hernia repair

Secondary outcomes, including postoperative complications, were assessed. Seven randomized controlled trials (1,108 patients) reported postoperative hematoma (Fig. 4). Glue fixation was associated with a lower hematoma rate compared with absorbable tacks (risk ratio 0.34; 95% CI 0.13–0.92; *p* = 0.03; I^2^ = 9%). However, this finding is uncertain given wide prediction intervals (0.06–1.80) and potential for false-positive results in TSA, indicating limited robustness of this finding.

**Fig. 5:**
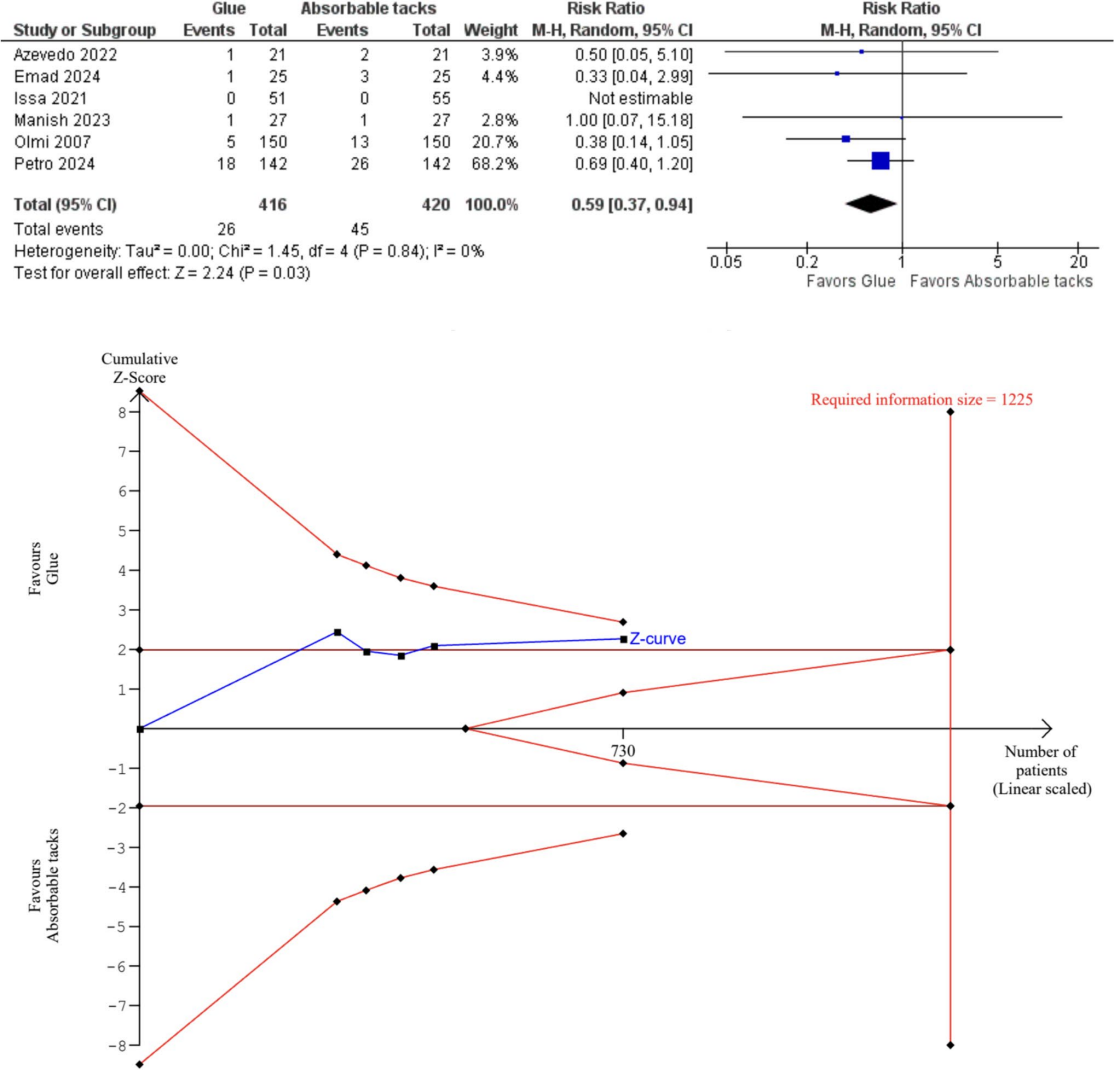
Forest plot and trial sequential analysis of postoperative seroma comparing glue fixation with absorbable tacks in laparoscopic inguinal hernia repair

Six randomized controlled trials (836 patients) reported postoperative seroma (Fig. 5). Glue fixation was associated with a lower seroma rate compared with absorbable tacks (risk ratio 0.59; 95% CI 0.37–0.94; *p* = 0.03; I^2^ = 0%). The 95% prediction interval (0.29–1.20) crossed unity, indicating uncertainty in the effect despite statistical significance in the pooled estimate. TSA suggested susceptibility to a false-positive finding, indicating that the observed effect may not be consistent across different clinical settings.

**Fig. 6:**
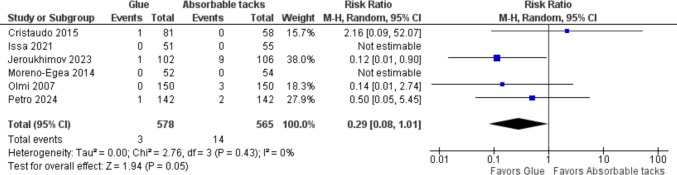
Forest plot of recurrence

Hernia recurrence was reported in six randomized controlled trials; however, only four studies documented at least one recurrence event (Fig. 6). Reported recurrence rates during the available follow-up were low in both groups (glue: 3/578 [0.5%] vs absorbable tacks: 14/565 [2.5%]), with no statistically significant difference observed (risk ratio 0.29; 95% CI 0.08–1.01; *p* = 0.05; I^2^ = 0%). TSA suggested a potential false-negative finding; however, the required information size was not reached, limiting the reliability of this result.

**Fig. 7:**
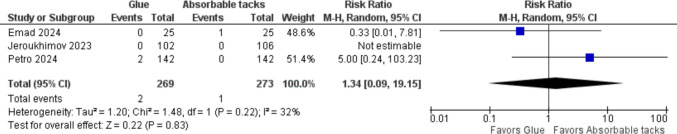
Forest plot of wound infection

**Fig. 8:**
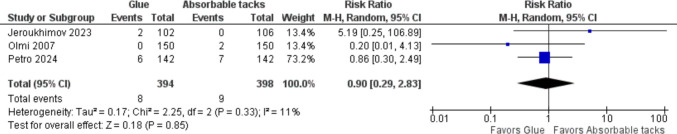
Forest plot of urinary retention

**Fig. 9:**
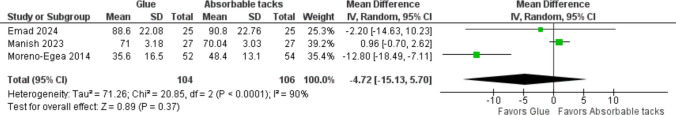
Forest plot of operative time

Wound infection and urinary retention were each reported in three randomized controlled trials (Figs. 7 and 8). No significant differences were observed between glue fixation and absorbable tacks for wound infection (risk ratio 1.34; 95% CI 0.09–19.15; *p* = 0.83; I^2^ = 32%) or urinary retention (risk ratio 0.90; 95% CI 0.29–2.83; *p* = 0.85; I^2^ = 11%). For both outcomes, TSA could not be reliably performed due to insufficient information size, with less than 1% of the required information, precluding meaningful interpretation. Operative time was reported in three randomized controlled trials (Fig. 9). No significant difference was observed between fixation methods (mean difference − 4.72 min; 95% CI − 15.13 to 5.70; *p* = 0.37), with substantial heterogeneity (I^2^ = 90%). TSA suggested insufficient evidence to detect a difference, although the required information size was not reached.

### Quality assessment

Four RCTs were single-blinded [9, 13–15], two reported a double-blind design [16, 17], one was unblinded [18], and blinding procedures were not reported in two studies [19, 20]. Across studies, concerns were also identified in domains related to deviations from intended interventions and outcome measurement, reflecting variability in study conduct.

**Fig. 10:**
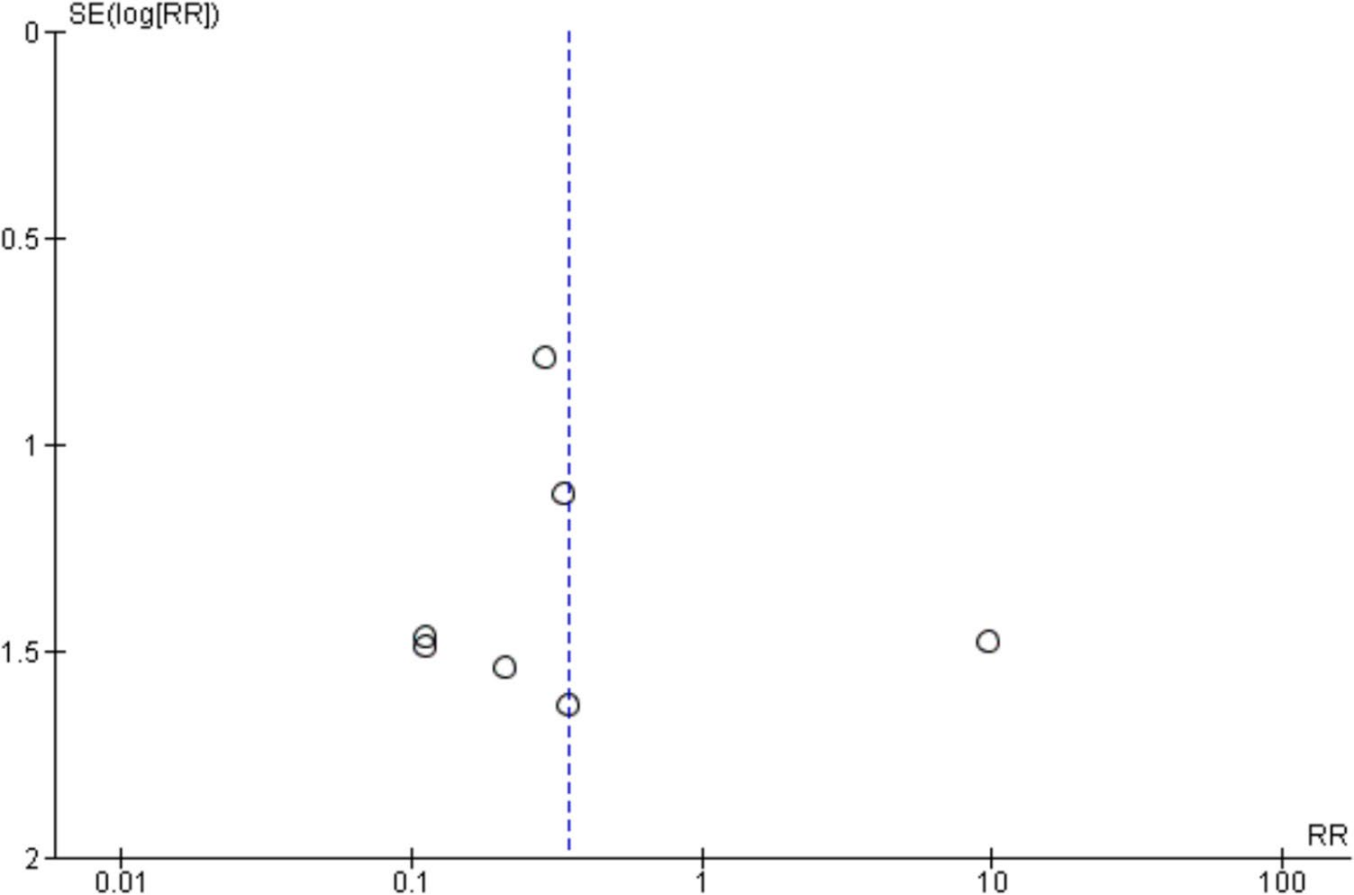
Funnel plot analysis of included studies

**Fig. 11:**
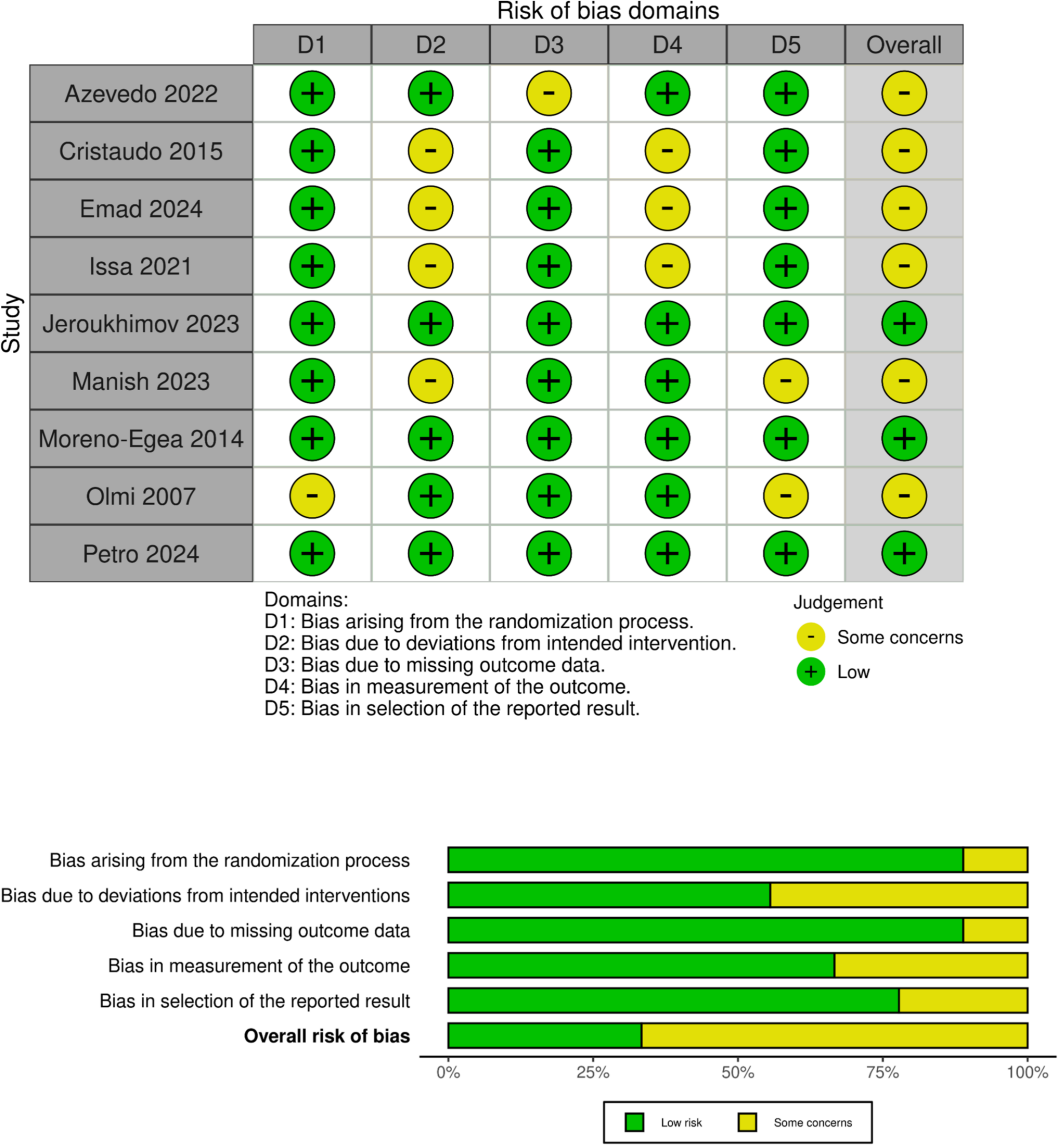
Risk of bias according to the Rob-2 tool

The funnel plot for hematoma, including seven studies, demonstrated an asymmetric distribution; however, given the limited number of studies, this finding should be interpreted with caution (Fig. 10). The risk of bias assessment is presented in Fig. 11.

### GRADE assessment

**Fig. 12:**
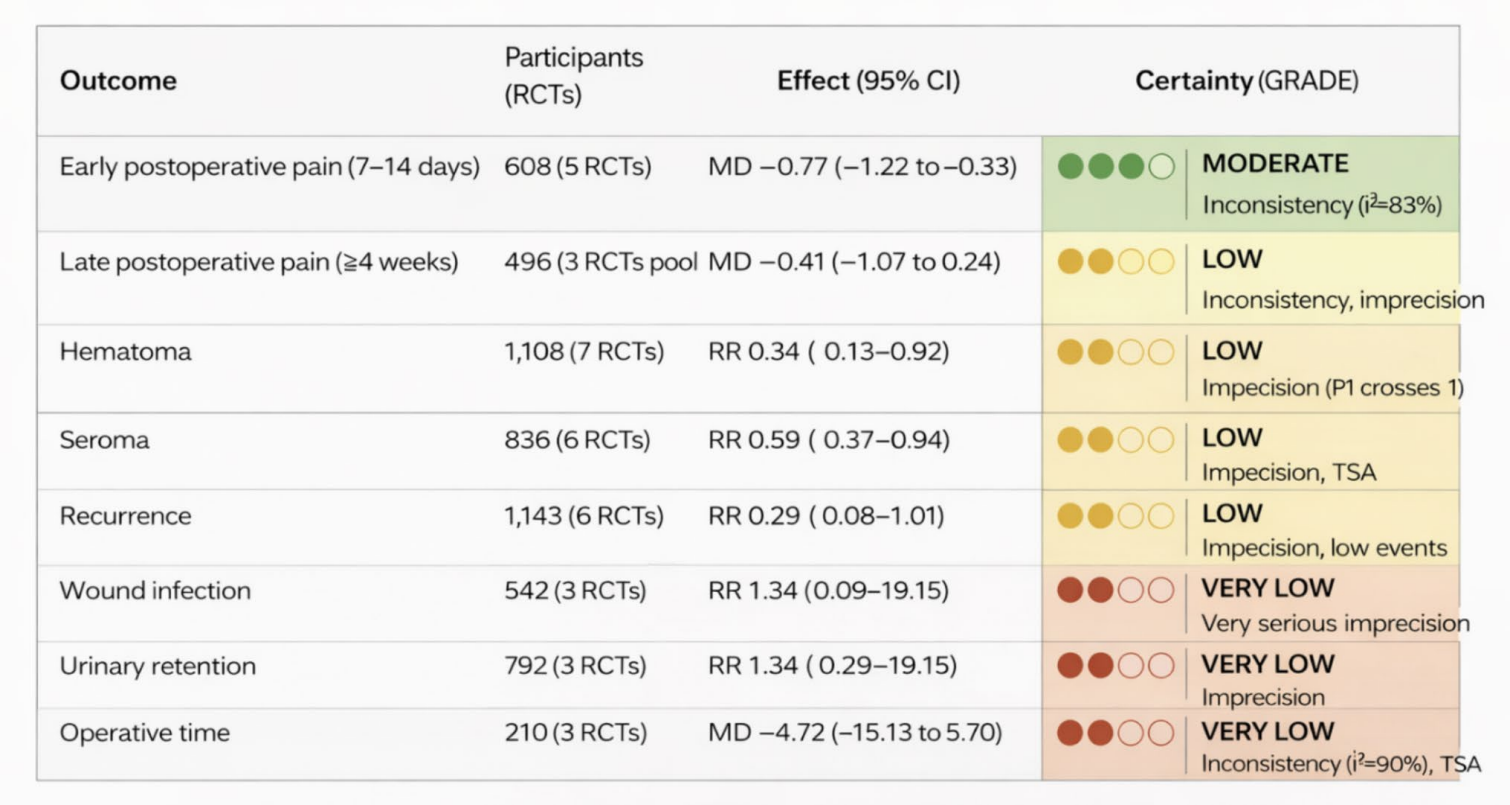
Certainty of evidence assessed using the GRADE approach for outcomes comparing glue versus absorbable tacks in laparoscopic inguinal hernia repair

Certainty of evidence across outcomes was assessed using the GRADE approach. Overall, the certainty of evidence was moderate for early postoperative pain, while all other outcomes were rated as low or very low due to inconsistency, imprecision, and insufficient information size as indicated by trial sequential analysis (Fig. 12), which limited the strength and clinical applicability of conclusions across most outcomes.

## Discussion

This systematic review and meta-analysis comprising nine RCTs and 1,289 patients compared glue versus AT as mesh fixation methods in LIHR. The main findings from the pooled analysis were: (1) available randomized evidence suggests that glue fixation may be associated with lower early postoperative pain during the first two postoperative weeks, whereas this advantage appears to diminish beyond four weeks; (2) glue fixation was associated with lower rates of hematoma and seroma, although these findings remain uncertain; (3) no statistically significant difference between groups for hernia recurrence rates, urinary retention, surgical site infection and operative time.

Importantly, these findings apply only to clinical scenarios in which mesh fixation is considered necessary, and do not inform the broader decision of fixation versus non-fixation, which remains the predominant strategy in contemporary practice. Although glue fixation demonstrated a significant reduction in early postoperative pain, the certainty of evidence was rated as moderate, mainly due to heterogeneity across studies. For other outcomes, including complications and recurrence, the certainty was low to very low, reflecting imprecision and insufficient information size despite pooled estimates suggesting potential benefit, which limits the strength of clinical conclusions for these outcomes.

Recent evidence has explored mesh fixation strategies for laparoscopic inguinal hernia repair, although important methodological limitations persist. Lin et al. (2025) demonstrated reduced postoperative pain with NB2C glue compared with sutures or tacks; however, their analysis combined open and laparoscopic approaches, lacked standardized pain assessment, and did not incorporate trial sequential analysis [21]. Similarly, Kitching et al. (2025) reported the benefits of glue over tackers using TSA but included both absorbable and non-absorbable devices, the latter being associated with increased postoperative pain, without isolating their specific effects [22]. In parallel, a narrative review by Rancke-Madsen et al. (2025) highlighted the substantial variability in fixation techniques and the lack of high-certainty evidence favouring a single approach. In contrast, our study exclusively included absorbable tacks, reducing clinical heterogeneity and enabling more precise comparisons [7]. Building upon the prior literature, our analysis was restricted to randomized controlled trials, focused on early postoperative pain, and incorporated TSA to control for random errors, alongside leave-one-out sensitivity analysis and prediction intervals to enhance robustness and external validity. Taken together, available randomized evidence suggests that glue fixation may be associated with lower early postoperative pain compared with absorbable tacks; however, uncertainty remains regarding the consistency, magnitude, and clinical applicability of this effect.

The primary findings of this study corroborate existing literature, demonstrating that pain scores were significantly lower in the glue group compared to conventional traumatic fixation methods. Kapris et al. [23] reported post-hernioplasty neuralgia rates of 0.5–14% with staple fixation, underscoring the potential for mechanical fixation to exacerbate nociceptive stimulation, compared with non-penetrating methods, which may be associated with local inflammatory responses [14]. Although the pooled analysis favoured glue between postoperative days 7 and 14, the wide prediction interval indicates variability across clinical settings. Trial sequential analysis suggested evidence of a potential effect; however, this should be interpreted cautiously given substantial heterogeneity (τ^2^ = 0.19; I^2^ = 83%) and variability not explained by sensitivity analysis. Nevertheless, pooled estimates suggested lower early postoperative pain with glue fixation, although the clinical relevance and consistency of this effect remain uncertain across settings.

In contrast, this study did not directly assess chronic postoperative pain, as only three trials reported follow-up beyond six months [24]. Accordingly, the early benefit observed with glue fixation was not maintained beyond four weeks. Trial sequential analysis and prediction intervals may suggest insufficient cumulative evidence rather than a suggested absence of effect. While prior data indicate differences between fixation methods in chronic pain [24–26], more recent evidence comparing absorbable tacks and fibrin glue suggests similar long-term outcomes [24], possibly due to advances in tack design and absorbability [26–28].

The literature indicates that the formation of seroma and hematoma is multifactorial, with the extent of dissection and duration of surgery being the most prevalent factors [29]. Pooled analyses suggested lower rates of hematoma and seroma with glue fixation compared with absorbable tacks; however, prediction intervals and trial sequential analysis indicated uncertainty and susceptibility to random error. This trend may in part reflect the atraumatic nature of glue fixation, which could be associated with reduced local tissue injury and inflammatory response. Moreover, it may limit damage to small intramuscular blood vessels, thereby decreasing hemorrhage and consequently, the formation of hematoma [30].

No statistically significant differences in hernia recurrence were observed between glue fixation and absorbable tacks. However, low event rates, limited follow-up, wide confidence and prediction intervals, and trial sequential analysis not reaching the required information size indicate that the evidence remains underpowered and inconclusive. A large Danish registry study with five-year follow-up similarly reported no difference between techniques, with recurrence rates of 5–8% [31]. These findings should be interpreted with caution, given the observational nature of the data and differences from randomized evidence.

Operative time did not differ between fixation methods, although wide uncertainty and trial sequential analysis suggested underpowered and inconclusive evidence [28]. Surgical site infection rates were also comparable, consistent with both approaches being clean procedures, but findings remain inconclusive. Likewise, urinary retention did not differ between groups, in line with previous reports [32], with trial sequential analysis indicating insufficient evidence rather than true equivalence.

Nonetheless, this study has some limitations. The relatively high heterogeneity observed in our findings regarding postoperative outcomes may be explained by several factors, including the limited number of included studies (*n* = 9), many with small sample sizes, as well as substantial variations in surgical protocols. There was also considerable variation in mesh materials, glue types, and fixation techniques across studies. Four studies used biologic glue and five used synthetic glue, while the number and placement of tacks were often not reported. In addition, clinically relevant variables such as defect size and indications for fixation were inconsistently described, limiting further assessment of potential subgroup effects and clinical applicability. To explore the robustness of these findings and identify potential sources of heterogeneity, a leave-one-out sensitivity analysis was performed. Additionally, postoperative pain was assessed at different time points across studies, impeding data harmonization, while restricted and inconsistent follow-up periods further limited comparability. These methodological discrepancies likely contributed to both the increased heterogeneity and the potential risk of bias in our pooled estimates, underscoring the need for standardized reporting in future surgical studies to improve the reliability and comparability of results.

Future studies should aim to improve methodological consistency and reporting standards to enhance comparability across studies, including clearer documentation of surgical techniques and fixation strategies when applied. Postoperative pain assessment would benefit from the use of validated and consistently applied measures, such as the Visual Analog Scale (VAS), at predefined time points. Further randomized controlled trials are warranted to better evaluate different fixation approaches, including variations in glue composition, and to strengthen the available evidence base.

## Conclusion

In this meta-analysis of nine randomized trials including 1,289 patients, glue fixation may be associated with lower early postoperative pain and a trend toward fewer hematoma and seroma events compared with absorbable tacks in laparoscopic inguinal hernia repair. However, these findings should be interpreted with caution, as prediction intervals and trial sequential analysis indicate variability across studies and susceptibility to random error, particularly for postoperative complications. No sustained difference in pain was observed beyond four weeks, and the available evidence remains insufficient to establish equivalence or exclude clinically relevant effects. Similarly, outcomes such as recurrence, wound infection, urinary retention, and operative time were infrequent and underpowered, limiting definitive conclusions. Overall, available randomized evidence suggests that glue fixation may be associated with lower early postoperative pain compared with absorbable tacks; however, uncertainty remains regarding the consistency, magnitude, and clinical applicability of this effect. Further well-designed randomized trials with standardized techniques and adequate follow-up are needed to clarify long-term outcomes and postoperative complications.

## Supplementary Information

Below is the link to the electronic supplementary material.

Supplementary file1 (DOCX 10 KB)

## Data Availability

Available upon request.
