# Supplementary material for: Glue versus absorbable tacks for mesh fixation in laparoscopic inguinal hernia repair: a systematic review and meta-analysis of postoperative pain and complications
Source: Hernia. 2026 Jul 3;30(1):279. doi: 10.1007/s10029-026-03757-w (PMC13331940; doi:10.1007/s10029-026-03757-w)
Supplement: Supplementary file 1 — Supplementary file1 (DOCX 10 KB) [file 10029_2026_3757_MOESM1_ESM.docx]

**Glue versus absorbable tacks for mesh fixation in laparoscopic inguinal hernia repair: a systematic review and meta-analysis of postoperative pain and complications**

**Supplementary Table 1.** Electronic search strategy and number of records retrieved from each database.

| **Database** | **Search strategy** | **Articles** |
| --- | --- | --- |
| Pubmed | (Hernia OR Hernorraphy OR Hernioplasty OR inguinal hernia OR groin hernia OR hernia repair) AND (Glue OR cyanoacrylate OR Tissue Adhesives OR Adhesive OR sealant OR sutureless OR “no suture” OR “no fixation”) AND (tack OR tacks OR tacker OR tackers OR taking OR staple OR staples OR stapling OR prosthesis fixation) | 580 |
| Cochrane | (Hernia repair OR herniorrhaphy OR hernioplasty OR inguinal hernia OR groin hernia OR femoral hernia) AND (Glue OR cyanoacrylate OR Tissue Adhesives OR Adhesive OR sealant) AND (tack OR tacks OR tacker OR tackers OR taking OR staple OR staples OR stapling OR prosthesis fixation) | 122 |
| Embase | (Hernia OR Hernorraphy OR Hernioplasty OR inguinal hernia OR groin hernia OR hernia repair) AND (Glue OR cyanoacrylate OR Tissue Adhesives OR Adhesive OR sealant OR sutureless OR “no suture” OR “no fixation”) AND (tack OR tacks OR tacker OR tackers OR taking OR staple OR staples OR stapling OR prosthesis fixation) | 191 |
